# Ruminal Fermentation Pattern, Bacterial Community Composition, and Nutrient Digestibility of Nellore Cattle Submitted to Either Nutritional Restriction or Intake of Concentrate Feedstuffs Prior to Adaptation Period

**DOI:** 10.3389/fmicb.2020.01865

**Published:** 2020-07-31

**Authors:** Ana C. J. Pinto, Gustavo P. Bertoldi, Luana D. Felizari, Evandro F. F. Dias, Breno L. Demartini, Ana B. C. P. Nunes, Mariana M. Squizatti, Antonio M. Silvestre, Lucas F. R. Oliveira, Joseph H. Skarlupka, Paulo H. M. Rodrigues, Gustavo D. Cruz, Garret Suen, Danilo D. Millen

**Affiliations:** ^1^School of Veterinary Medicine and Animal Science, São Paulo State University (UNESP), Botucatu, Brazil; ^2^College of Technology and Agricultural Sciences, São Paulo State University (UNESP), Dracena, Brazil; ^3^Department of Bacteriology, University of Wisconsin–Madison, Madison, WI, United States; ^4^School of Veterinary Medicine and Animal Science, University of São Paulo, São Paulo, Brazil; ^5^Purina Animal Nutrition LLC, Arden Hills, MN, United States

**Keywords:** adaptation, feedlot, microbiota, Nellore, rumen

## Abstract

Beef cattle are key contributors to meat production and represent critical drivers of the global agricultural economy. In Brazil, beef cattle are reared in tropical pastures and finished in feedlot systems. The introduction of cattle into a feedlot includes a period where they adapt to high-concentrate diets. This adaptation period is critical to the success of incoming cattle, as they must adjust to both a new diet and environment. Incoming animals are typically reared on a variety of diets, ranging from poor quality grasses to grazing systems supplemented with concentrate feedstuffs. These disparate pre-adaptation diets present a challenge, and here, we sought to understand this process by evaluating the adaptation of Nellore calves raised on either grazing on poor quality grasses (restriction diet) or grazing systems supplemented with concentrate (concentrate diet). Given that nutrient provisioning from the diet is the sole responsibility of the ruminal microbial community, we measured the impact of this dietary shift on feeding behavior, ruminal fermentation pattern, ruminal bacterial community composition (BCC), and total tract digestibility. Six cannulated Nellore bulls were randomly assigned to two 3 × 3 Latin squares, and received a control, restriction, or concentrate diet. All cohorts were then fed the same adaptation diet to mimic a standard feedlot. Ruminal BCC was determined using Illumina-based 16S rRNA amplicon community sequencing. We found that concentrate-fed cattle had greater dry matter intake (*P* < 0.01) than restricted animals. Likewise, cattle fed concentrate had greater (*P* = 0.02) propionate concentration during the adaptation phase than control animals and a lower Shannon’s diversity (*P* = 0.02), relative to the restricted animals. We also found that these animals had lower (*P* = 0.04) relative abundances of *Fibrobacter succinogenes* when compared to control animals during the pre-adaptation phase and lower abundances of bacteria within the *Succinivibrio* during the finishing phase, when compared to the control animals (*P* = 0.05). Finally, we found that animals previously exposed to concentrate were able to better adapt to high-concentrate diets when compared to restricted animals. Our study presents the first investigation of the impact of pre-adaptation diet on ruminal BCC and metabolism of bulls during the adaptation period. We suggest that these results may be useful for planning adaptation protocols of bulls entering the feedlot system and thereby improve animal production.

## Introduction

Meat and its derivative products are essential components of the human diet as a source of energy and protein. Beef cattle are important sources of meat and their production is ubiquitous across the globe (Hocquette et al., 2018; Ritch and Roser, 2019). As the human population continues to expand, it is predicted that the demand for beef as a protein source will also increase (Smith et al., 2018), and efforts are needed to improve the efficiency of beef cattle production.

Beef production systems, such as those in Brazil, utilize feedlots, which source beef calves from smaller farms and then maintain these cattle on a high concentrate finishing diet. In Brazil, the Nellore is one of the most predominantly reared beef cattle breeds, primarily due to its ability to readily thrive on low quality tropical grasses. Efforts to improve performance during the finishing period include dietary addition of rapidly fermentable carbohydrates, which can promote the growth of lactate-producing ruminal bacteria and lead to health problems such as ruminal acidosis (Pacheco et al., 2012; Millen et al., 2015). As a result, numerous nutritional strategies have been adopted to control for issues such as ruminal acidosis including the use of additives to eliminate undesirable ruminal microorganisms and the gradual introduction of non-fibrous carbohydrates into the diet.

Previous work has demonstrated that the ability of steers to respond productively to the finishing diet is dependent on their success during the adaptation period, which spans the time from entry into the feedlot system through to the introduction of a high concentrate finishing diet. During this period, the bull calf is required to transition from a forage-based diet to a high-concentrate diet. In general, the average adaptation period in Brazilian feedlots is reported to be 16.2 days (Pinto and Millen, 2018). Further work evaluating different adaptation periods (Perdigão et al., 2017; Barducci et al., 2019; Parra et al., 2019) suggested that Nellore cattle should spend a minimum of 14 days adapting to a high-concentrate diet in a step-up manner, where increasing amounts of concentrate are successively added to the adaptation diet. However, the application of these guidelines is confounded by the dietary history of the incoming calves, which can differ significantly by sourcing location. This includes farms that maintain their calves on a pre-adaptation diet ranging from grazing on poor quality tropical grasses to grazing systems supplemented with varying amounts of concentrate.

Regardless of nutritional background, all cattle arriving at Brazilian feedlots are adapted in the same way to a high-concentrate diet during the adaptation period. This can present problems, such as the aforementioned ruminal acidosis, during the finishing period and negatively impact animal health and productivity. For example, the inclusion of small amounts of grain (10 to 20%) in the pre-adaptation diet prior to feedlot arrival may trigger ruminal acidosis by ensuring that adequate numbers of amylolytic microorganisms, such as those from the genus *Succinivibrio*, are present to ferment the carbohydrates in the adaptation and finishing diet (Nagaraja and Titgemeyer, 2007; Nagaraja, 2016; Ren et al., 2019). Cattle that are nutritionally restricted to grazing on poor-quality tropical grasses prior to intake of a high-concentrate diet may respond negatively to the rapid influx of concentrate present in the adaptation diet and develop ruminal acidosis.

Little information is known about the effects of nutritional background on bacterial community composition (BCC) and ruminal fermentation patterns during the transition from a forage-based to concentrate-rich diet. Therefore, given the paucity of data regarding the impact of the pre-adaptation diet on the adaptation period in Nellore beef cattle, we conducted a study on beef cattle cohorts maintained on different diets and monitored their ruminal microbiota and metabolism during adaptation to a high-concentrate diet and finishing. In a previous study, Pereira et al. (2020) reported that cattle consuming concentrate feedstuffs prior to the adaptation phase improved feedlot performance, and we therefore hypothesized that cattle maintained on nutritionally restricted or grazing with concentrate feedstuffs during the pre-adaptation period would have different ruminal microbiota resulting in differences in both ruminal fermentation patterns and nutrient digestibility. The results from this work will be useful to producers by providing insights into the appropriate adaptation diet that should be provided to newly received cattle based on their previous diet history in order to minimize downstream adverse health effects and increase productivity.

## Materials and Methods

All procedures involving the use of animals in this study were in accordance with the guidelines established by the São Paulo State University Ethical Committee for Animal Research (protocol number 05/2015).

### Animals, Treatments, and Management

This trial was conducted at the São Paulo State University feedlot, Dracena Campus, Brazil. Six 20-month-old yearling Nellore bulls (236 ± 20 kg) fitted with ruminal cannulas were randomly assigned to a replicated 3 × 3 Latin square design. Inside each Latin square, animals received one of three pre-adaptation dietary treatments (Table 1) for a period of 14 days: Control (Tifton hay fed *ad libitum* + supplement), Restriction (Tifton hay fed at 1.4% of body weight + supplement); and Concentrate (Tifton hay fed *ad libitum* + 0.5% of body weight of a mix of concentrate feedstuffs and supplement). This was followed by an adaptation period consisting of 2 adaptation diets, which contained 72 and 79% concentrate (Supplementary Table S1) fed *ad libitum* for 6 days each (from day 15 to day 26). The finishing diet, containing 86% concentrate (Supplementary Table S1), was then fed from day 27 to day 33. We note that the experimental diets fed to cattle during the pre-adaptation, adaptation, and finishing phases were formulated according to the Large Ruminant Nutrition System (Fox et al., 2004) and was composed of sugarcane bagasse, Tifton hay, cracked corn grain, cottonseed meal, urea, limestone, and a mineral supplement.

**TABLE 1:** Feed ingredients and chemical composition of the experimental diets fed to cannulated Nellore cattle during the pre-adaptation phase.

**Treatments**	Control*	Restriction**	**Concentrate**
*Ingredients,% of dry matter* (*DM*)			
Tifton hay	97.38	97.38	79.44
Finely ground corn grain	–	–	16.67
Cottonseed meal (38% CP)	–	–	1.56
Urea	0.75	0.75	0.67
Supplement^1^	1.88	1.88	1.67
**Nutrient Content**			
Total digestible nutrients,% of DM	46.00	46.00	53.00
Net energy for maintenance, Mcal/kg of DM	1.31	1.31	1.59
Net energy for gain, Mcal/kg of DM	0.73	0.73	0.99
Crude protein,% of DM	12.40	12.40	12.50
Neutral detergent fiber (**NDF**),% of DM	73.50	73.50	62.90
Ether extract,% of DM	2.20	2.20	2.40
Physically effective NDF,% of DM	70.00	70.00	58.00
Ca,% of DM	0.58	0.58	0.53
P,% of DM	0.24	0.24	0.27

^1^Ca: 9.80%; P: 4.50%; Mg: 4.40%; K: 6.15%; Na: 11.45%; Cl: 6.60%; S: 4.00%; Co: 48.50 ppm; Cu: 516 ppm; Fe: 30 ppm; Mn: 760 ppm; Se: 9 ppm; Zn: 2516.5 ppm; and sodium monensin: 2000 ppm; *Control animals were fed Tifton hay at 1.8% of BW. **Restricted animal were fed Tifton hay at 1.4% of BW.

Nellore yearling bulls were housed in individual pens (72 m^2^) equipped with individual 6-m feed bunks, and free-choice water access to a water trough (3.00 × 0.80 × 0.20 m) shared by two animals. Yearling bulls were fed *ad libitum* once a day at 0800 h, and the dry matter intake (DMI) was calculated daily by weighing ration offered and orts, before the next morning delivery, and expressed both in kilograms and as a percentage of body weight. The amount of feed offered was adjusted daily based on the amount of orts left before morning feed delivery (0700 h). Body weight was measured at the beginning (day 1) and at the end (day 33) of each period at 0700 h.

### Feeding Behavior and Particle Sorting

Cattle were visually observed to evaluate feeding behavior every 5 min over three periods of 24 h. These observations were performed on days 4 (pre-adaptation phase), 15 (adaptation phase), and 26 (finishing phase) of the study, according to Robles et al. (2007). Feeding behavior data were recorded for each animal as follows: time spent eating, ruminating and resting (expressed in minutes), and number of meals per day. A meal was considered the non-interrupted time cattle spent in the feed bunk eating the ration. DMI was also measured for each animal on the days when feeding behavior data was collected. The meal length, in minutes, was calculated by dividing time spent eating by number of meals per day. The DMI per meal, in kilograms, was calculated by dividing DMI by the number of meals per day. In addition, the eating rate of dry matter (DM; time spent eating/DMI) and rumination rate of DM (time spent ruminating/DMI), both expressed in minutes per kilogram of DM, were calculated according to Pereira et al. (2015). Samples of diets and orts were collected for chemical analysis of neutral detergent fiber (NDF; Van Soest et al., 1991) to determine the intake of NDF on the day of feeding behavior data collection. Eating and rumination rates of NDF were calculated, as described above for DM, according to Pereira et al. (2015).

On the days of feeding behavior evaluation, samples of diets and orts were also collected for determination of particle-size distribution, which was performed by sieving using a Penn State Particle Size Separator and reported on an as-fed basis, as described by Heinrichs and Kononoff (1996). Particle sorting was determined as follows: n intake/n predicted intake, in which *n* = particle fraction screens of 19 mm (long), 8 mm (medium), 1.18 mm (short), and a pan (fine). Particle sorting values equal to 1 indicate no sorting, those <1 indicate selective refusals (sorting against), and those >1 indicate preferential consumption (sorting for; Leonardi and Armentano, 2003).

### Sample Collection and Laboratory Methods

#### *In situ* Degradability

Determination of *in situ* degradability was performed on days 6, 7, and 8 (pre-adaptation phase), 17, 18, and 19 (adaptation phase), and 29, 30, and 31 (finishing phase) of each experimental period using the nylon bag technique described by Mehres and Ørskov (1977). About 10 *g* of diet samples, previously dried at 65°C for 72 h, were weighed into 10 × 19 cm nylon bags with a pore size of 50 μm. Nylon bags were inserted into the rumen and incubated for 24 h. Immediately after their retrieval, bags were placed in a bucket with cold tap water to stop microbial fermentation, manually washed under cold tap water, and then oven dried at 65°C for 48 h. Samples not incubated in the rumen were also washed as described above. Samples were analyzed for DM (method 934.01; AOAC, 1990); crude protein (CP), by total N determination using the micro-Kjeldahl technique (method 920.87; AOAC, 1990); ether extract, determined gravimetrically after extraction using petroleum ether in a Soxhlet extractor (method 920.85; AOAC, 1990); and NDF (with heat stable α-amylase) and acid detergent fiber (ADF) according to Van Soest et al. (1991). Starch analysis was performed according to Pereira and Rossi (1995), with previous extraction of soluble carbohydrates, as proposed by Hendrix (1993). Ground samples were then ashed at 450°C in a muffle oven for determination of ash (AOAC, 1995) and organic matter (OM) concentration (method 924.05; AOAC, 1990). The apparent coefficient of nutrient degradability was calculated by the following equation: 100 × [01 – (bag weight after incubation – empty bag weight)/(bag weight before incubation – empty bag weight)].

#### Ruminal Fermentation Variables

Ruminal pH was continuously measured on days 5 (pre-adaptation phase), 16 (adaptation phase), and 27 (finishing phase) of each period for 24 h using a data logger pH (Model T7-1 LRCpH, Dascor^®^, Escondido, CA, United States; Penner et al., 2006). The indwelling electrode measured and recorded the ruminal pH every 10 min over the measurement period. Each electrode was standardized using pH 4.0 and 7.0 standards at the beginning and end of each session. The pH data were recorded as mean, maximum, and minimum pH, the area under the curve, and duration of time in which pH was below 6.2, 6.0, and 5.8. The area under the curve was calculated by multiplying the absolute value of deviations in pH by the time (min) spent below the established threshold for each measure divided by 60 and expressed as pH unit × h. Likewise, data loggers recorded rumen temperature and ox-redox potential (Penner et al., 2006). Ruminal fluid samples were collected at day 5 (pre-adaptation phase), 16 (adaptation phase), and 27 (finishing phase) of each period through the ruminal cannula with a vacuum pump at 0, 4, 8, and 12 h after the morning meal. Approximately 500 mL of rumen fluid was collected, at each sampling time, from 3 different parts of the rumen. A fraction of 100 mL of ruminal fluid was centrifuged at 2,000 × *g* for 20 min at room temperature, and 2 mL of the supernatant was added to 0.4 mL of formic acid and frozen at –20°C for further short-chain fatty acid (SCFA) analyses according to Erwin et al. (1961). The SCFAs acetate, propionate, and butyrate, were measured by gas chromatography (Finnigan 9001, Thermo Scientific, West Palm Beach, FL, United States) using a glass column 1.22 m in length and 0.63 cm in diameter packed with 80/120 Carbopack B-DA/4% (Supelco, Sigma-Aldrich, St. Louis, MO, United States). For NH3-N concentration determination, 2 mL of the supernatant was added to 1 mL of 1 N of H_2_SO_4_ solution and the centrifuge tubes were immediately frozen until the colorimetric analyses, according method described by Kulasek (1972), and adapted by Foldager (1977).

#### Ruminal Protozoa Counting

For total and differential counts of ruminal protozoa, samples of 10 mL of ruminal contents were collected on day 5 (pre-adaptation phase), 16 (adaptation phase), and 27 (finishing phase) of each period through the ruminal cannula with a vacuum pump at 4 h after the morning meal and stored in glass vials with 20 mL of 18.5% formaldehyde. Subsequently, the sample was stained with two drops of 2% brilliant green and diluted. Protozoa were identified (genus *Isotricha*, *Dasytricha*, *Entodinium*, and *Diplodiniinae* subfamily) and counted using a Neubauer Improved Bright-Line counting chamber (Hausser Scientific Partnership^®^, Horsham, PA, United States) by optical microscopy (Olympus CH-2^®^, Japan; Dehority, 1993).

#### Ruminal Bacterial Community Composition

Samples of 50 mL of whole rumen contents were collected on day 5 (pre-adaptation phase), 16 (adaptation phase), and 27 (finishing phase) of each period through the ruminal cannula at 4 h after the morning meal and stored at −80°C. After thawing, samples were separated by phase (liquid and solid). Proportionate amounts of liquid and solid phase rumen content, as determined by when the rumen was completely emptied and phases separated and measured, were combined and then processed to isolate DNA following the procedure detailed in Weimer et al. (2017).

The resuspended pellets were then processed to isolate DNA following the bead-beating method described by Weimer et al. (2017). The DNA was resuspended in 10 mM Tris HCl with 1 mM EDTA (pH 8.0), quantified fluorometrically using a Qubit (Invitrogen, Carlsbad, CA, United States), and stored at 4°C before preparation of the DNA library. Universal primers amplifying the Variable 4 region of the bacterial 16S rRNA gene were used to perform PCR (F- GTGCCAGCMGCCGCGGTAA; R- GGACTACHVGGGTWTCTAAT), as described by Kozich et al. (2013). The primers also included unique barcodes for multiplexing and adapters suitable for sequencing using Illumina technology (F- AATGATACGGCGACCACCGAGATCTACAC; R- CAAGCAGAAGACGGCATACGAGAT; Kozich et al., 2013). The PCR reactions contained 25–50 ng of DNA, 10 μM of each primer, 12.5 μL of 2X KAPA HotStart ReadyMix (KAPA Biosystems, Wilmington, MA, United States), and water to a total volume of 25 μL. Cycling conditions were as follows: initial denaturation of 95°C for 3 min, 25 cycles of 95°C for 30 s, 55°C for 30 s, and 72°C for 30 s, and a final extension at 72°C for 5 min. Gel electrophoresis was performed using a 1.0% low-melt agarose gel (National Diagnostics, Atlanta, GA, United States), where bands present at ∼380 bp indicated successful amplification.

Bands were excised from the gel and DNA was extracted from the bands using a ZR-96 Zymoclean Gel DNA Recovery Kit (Zymo Research, Irvine, CA, United States). No-template negative controls were included for each set of PCRs, and absence of a band in the gel indicated no contamination was present. Extracted DNA was quantified in duplicate on 96-well microplates according to manufacturer’s instructions for the Quant-iT dsDNA Broad-Range Assay Kit, using reagents from a Qubit dsDNA Assay Kit (Thermo Fisher Scientific, Waltham, MA, United States), read on a Synergy 2 Multi-Mode Reader (BioTek, Winooski, VT, United States) after a programmed 3-s shaking period and a 2-min incubation at 22°C. The extracted DNA was equimolar pooled. The final library was sequenced using a MiSeq v2 2 × 250 kit (Illumina, San Diego, CA, United States), with a final library concentration of 10 pmol/L and 10% PhiX control. Custom sequencing primers as described by Kozich et al. (2013) were used. Sequences were demultiplexed according to their sample-specific indices on the Illumina MiSeq. The sequences used in this study were deposited into the National Center for Biotechnological Information’s Short Read Archive and is available under BioProject Accession PRJNA641164.

The program *mothur* (v. 1.41.1) was used for further processing (Schloss et al., 2009). Paired-end sequences were combined to form contigs and poor-quality sequences were removed (e.g., elimination of sequences with ambiguous base pairs, homopolymers greater than 8 bp, and sequences shorter than 200 bp). The SILVA 16S rRNA gene reference alignment database (v132; Quast et al., 2013) was used to screen for alignment to the correct region. Pre-clustering was performed (diffs = 2) to reduce error and chimeras were detected and removed using UCHIME (Edgar et al., 2011). The GreenGenes database (DeSantis et al., 2006), August 2013 release, was used to classify sequences with a bootstrap value cutoff of 80. Sequences classified to cyanobacteria, mitochondria, Eukarya, or Archaea were removed. Singletons were removed to streamline analysis.

### Total Tract Apparent Digestibility

From days 5–13 and 24–32 of each experimental period, titanium dioxide was used as an external marker to estimate total tract apparent nutrient digestibility (Pezzato et al., 2002). The marker was added at 1 g/kg of diet DM through ruminal cannula. Samples of feces, diets, and orts were collected from day 10 to 14 (pre-adaptation phase), and from day 29 to 33 (finishing phase) twice a day. Samples were composited by phase (approximately 200 *g*). Feed, orts, and fecal samples were dried at 55°C for 72 h and ground to pass a 1-mm screen. Composite samples per animal per period were used to determine DM (method 934.01; AOAC, 1990); CP (method 920.87; AOAC, 1990); ether extract (method 920.85; AOAC, 1990); NDF (with heat stable α-amylase); and ADF according to Van Soest et al. (1991), starch (Pereira and Rossi, 1995), ash (AOAC, 1995), and OM concentration (method 924.05; AOAC, 1990), as described above for *in situ* degradability of nutrients.

### Ruminal Dynamics

The ruminal dynamics were assessed by total rumen emptying. The ruminal digesta was manually removed from each animal through rumen cannula to determine the disappearance rate in the rumen as described by Dado and Allen (1995). On days 9 (pre-adaptation phase), 20 (adaptation phase), and 32 (finishing phase) of each experimental period, the emptying was carried out at 1100 h, 3 h after delivering the morning meal, when the rumen is theoretically at the highest level of volume. The same procedure was performed on days 10 (pre-adaptation phase), 21 (adaptation phase), and 33 (finishing phase) of each experimental period at 0800 h, immediately before the delivery of the morning meal, when the rumen is theoretically at its lowest volume. During the withdrawal of whole ruminal contents, the liquid and solid phases were separated with the aid of sieve and bucket and weighed. The solid and liquid phases were manually homogenized and 1 kg samples were taken for determination of DM. Immediately afterward, the digesta was reconstituted and placed back in the rumen of animals. The rumen pool of DM and its disappearance rate was calculated based on the dry weight of each sample (55°C for 72 h). The DM disappearance rate was considered equal to intake rate, and they were estimated using the formula (Robinson et al., 1987):

DMdisappearancerate(%/h)

=Daily⁢DM⁢intake⁢(kg)/DM⁢Ruminal⁢contents⁢(kg)/24

### Statistical Analysis

Results were analyzed by SAS software (SAS Inst. Inc., Cary, NC, United States), and tests for normality (Shapiro–Wilk’s and Kolmogorov–Smirnov’s) and heterogeneity of treatment variances (GROUP option of SAS) were performed before analyzing the data. Feed intake, feeding behavior, particle sorting, rumen pH variables, *in situ* degradability, total tract digestibility and ruminal dynamics variables were analyzed by MIXED procedure of SAS. The model included the effects of treatments as fixed factors. The effects of period, square, period × square, square × treatments, animal nested within square, and period × animal nested within square were considered random factors. The variables total concentration and molar proportion of SCFA and NH3-N concentration were analyzed by MIXED procedure of SAS with repeated measures (Littell et al., 1998). The model accounted for the same effects as described above plus time and its interactions with treatments. Each variable analyzed as repeated measures was subjected to 8 covariance structures: unstructured, compound symmetric, heterogeneous compound symmetric, autoregressive of order one [AR(1)], heterogeneous first-order autoregressive [ARH(1)], toeplitz, heterogeneous toeplitz, and ante-dependence of order one [ANTE(1)]. The covariance structure that yielded the smaller Akaike and Schwarz’s Bayesian criterion based on their −2 res log likelihood was considered to provide the best fit. Results were considered significant at *P* < 0.05 level. Effects were considered significant at *P* ≤ 0.05. All means presented are least squares means, and effects were separated by PDIFF option of SAS.

Bacterial sequences were grouped into operational taxonomic units (OTUs) at 97% sequence similarity. Good’s coverage (Good, 1953) was calculated in *mothur* for all samples and a Good’s coverage ≥ 0.95 was considered to have sufficient sequencing depth. The OTU counts were normalized to 10,000 sequences per sample, and the normalized counts of OTUs by sample were used for further analysis. Alpha diversity (community diversity within individual animals within each period) was assessed using Chao’s (1984) estimate of species richness and Shannon’s (2001) diversity index. Differences in community diversity and richness between animals were assessed by overall 2-way ANOVA in R v3.2.1 (R Core Team, 2011). Beta diversity (differences in community composition between samples) was assessed by using non-metric multidimensional scaling to visualize differences between samples calculated using the Bray–Curtis dissimilarity metric (Bray and Curtis, 1957). Changes in total community structure (relative abundance, Bray–Curtis metric) were assessed using permutational multivariate ANOVA (PERMANOVA) in R (*vegan* package; v 2.5-2 (Oksanen et al., 2019). Pairwise comparisons between each group were quantified PERMANOVA, and *P*-values were FDR-corrected.

To look for interactions between protozoal counts and pH measurements, the *glm* function in the *stats* package in R was used. The distribution (*family* =) for the command that best displayed no pattern in a Residuals vs. Fitted graph, and a normal distribution of residuals was used for each comparison; if not specified, the default *family* for *glm()* was used. For interactions between specific members of the BCC and measured metrics (protozoal counts, pH, and VFA measurements), the *cor* command in the *stats* package in R was first used to identify correlations using Kendall’s tau correlation method (*method* = *”kendall”*; Kendall, 1938). Before using the *cor* command, an abundance cutoff was used: any OTU which did not have more than 2 sequences in any one sample was removed from the dataset. Only OTUs with a strong correlation score (−0.50 ≤ *r* ≥ 0.50) were considered for further analysis. Each OTU was tested against the metric of interest using the *glm* function, with a *P-*value < 0.05 considered to indicate a significant interaction.

## Results and Discussion

Six 20-month-old yearling Nellore bulls (236 ± 20 kg) fitted with ruminal cannulas were randomly assigned to a replicated 3 × 3 Latin square design. Inside each Latin square, animals received one of three treatments during the pre-adaptation period of 14 days: a control diet, a restriction diet, and a concentrate diet. All animals received a high-concentrate diet for 12 days during the adaptation period, followed by a finishing diet for 7 days (Table 1 and Supplementary Table S1). We found that when we averaged intakes collected daily during the pre-adaptation phase, concentrate-fed cattle had greater (*P* < 0.01) DM intake than the control group (5.51 vs. 5.15 kg), which had greater intake then the restricted diet group (3.87 kg; data not shown). Likewise, during the adaptation phase, cattle receiving concentrate had greater (*P* < 0.01) DM intake than cattle from either the control or restriction groups (7.91 kg vs. 7.41 and 7.20 kg, respectively; data not shown). Finally, during the finishing phase, cattle from the restriction group presented lower (*P* < 0.01) DM intake than cattle on either the control or concentrate fed groups (8.82 kg vs. 9.32 and 9.17 kg, respectively; data not shown).

Our finding that cattle consuming concentrate in the pre-adaptation phase had greater (*P* < 0.01) DM intake during the adaptation period than either the control or restriction group suggests that low amounts of concentrate prior to the adaptation phase favors cattle adaptation. This is concomitant with our finding that, during the adaptation phase, concentrate-fed cattle had lower concentrations of ammonia (*P* < 0.05) and greater concentrations of SCFAs in the rumen (*P* = 0.02), including acetate (*P* = 0.01), and propionate (*P* = 0.04; Table 2), relative to the control group, without negatively impacting (*P* > 0.05) ruminal pH (Supplementary Table S2). In addition, the greater solid (*P* = 0.05) and DM (*P* = 0.03) disappearance rate in the concentrate group (Supplementary Table S3) during adaptation indicates that a greater amount of DM was either degraded in the rumen or bypassed the rumen-reticulum altogether.

**TABLE 2:** Effects of either nutritional restriction or intake of concentrate feedstuffs during the phases of pre-adaptation (day 5), adaptation (day 16), and finishing (day 27) on ruminal short-chain fatty acids and ammonia concentrations in cannulated Nellore cattle.

	**Treatment (Trt)**			**Time after feeding**					***P*-value**		
**Item**	**Control**	**Restriction**	**Concentrate**	**0 h**	**4 h**	**8 h**	**12 h**	**SEM**	**Trt**	**Time**	Trt*Time
**Pre-adaptation (day 5)**											
Total SCFA^1^, m*M*	87.91^a^	81.80^b^	87.64^a^	80.00	77.76	87.84	97.53	2.73	<0.01	<0.01	0.17
Acetate, mol/100 mol	66.57^a^	61.48^b^	64.38^a^	60.88	58.09	65.23	72.39	1.75	0.04	<0.01	0.07
Propionate, mol/100 mol	14.61^ab^	14.18^b^	15.30^a^	13.45	13.38	15.10	16.88	0.77	<0.01	<0.01 (L)	0.11
Butyrate, mol/100 mol	6.73	6.14	7.95	5.67	6.29	7.51	8.28	0.37	<0.01	<0.01	<0.01
Acetate:Propionate	4.60	4.38	4.26	4.56	4.38	4.37	4.34	0.10	0.07	<0.01	0.02
Ammonia, m*M*	4.44	5.25	5.91	5.11	10.09	2.51	3.10	0.62	<0.01	<0.01	<0.01
**Adaptation (day 16)**											
Total SCFA^1^, m*M*	99.69^b^	103.49^a^	105.16^a^	94.72	99.53	104.41	112.46	3.36	0.02	<0.01 (L)	0.36
Acetate, mol/100 mol	66.09^b^	68.63^a^	69.80^a^	63.84	66.46	69.01	73.36	1.95	0.01	<0.01 (L)	0.36
Propionate, mol/100 mol	22.45^b^	24.44^ab^	25.47^a^	21.25	22.73	24.88	27.63	1.14	0.04	<0.01	0.12
Butyrate, mol/100 mol	11.15	10.42	9.90	9.63	10.34	10.51	11.47	0.68	0.24	0.02 (L)	0.80
Acetate:Propionate	3.00	2.86	2.82	3.05	2.97	2.83	2.72	0.10	0.36	<0.01	0.01
Ammonia, m*M*	21.31^a^	21.17^a^	18.48^b^	12.64	24.06	20.55	24.02	0.99	0.05	<0.01(C)	0.10
**Finishing (day 27)**											
Total SCFA^1^, m*M*	102.51	100.26	101.75	102.71	97.76	97.95	107.58	3.51	0.78	<0.01 (Q)	0.27
Acetate, mol/100 mol	62.20	63.57	63.70	64.48	60.62	61.00	66.52	1.56	0.83	<0.01 (Q)	0.27
Propionate, mol/100 mol	28.34^a^	25.09^b^	25.89^ab^	25.46	25.43	25.73	29.14	2.67	0.05	<0.01 (L)	0.29
Butyrate, mol/100 mol	11.97	11.60	12.16	12.77	11.72	11.23	11.92	0.74	0.60	<0.01 (L)	0.62
Acetate:Propionate	2.30	2.67	2.70	2.73	2.57	2.52	2.40	0.23	0.09	0.04 (L)	0.99
Ammonia, m*M*	20.40	21.23	20.93	12.20	22.98	21.99	26.24	1.82	0.60	<0.01 (L)	0.08

*^1^Short-chain fatty acids. ^*a*,*b*^For treatment effect, within a row means without common superscript letter differ (*P* < 0.05).*

We found that the control and restriction groups likely require a longer passage time through the rumen, relative to the concentrate-fed group, in order to degrade DM, which negatively impacted DM intake during the adaptation phase. We note that during the adaptation phase, animals from the restriction group sorted for long (*P* = 0.05) and medium (*P* < 0.01) diet particles and against fine (*P* = 0.05) diet particles (Supplementary Table S4). This is likely due to ruminal acidification as they also increased (*P* = 0.02) production of total SCFA concentration, relative to the control group (Table 2). In contrast, cattle reared on a restricted diet during the pre-adaptation phase decreased (*P* = 0.05) DM intake per meal during the adaptation phase, when compared to concentrate-fed animals (Supplementary Table S5), which had greater ruminal degradability of DM and starch (*P* = 0.05; Table 3).

**TABLE 3:** Effects of either nutritional restriction or intake of concentrate feedstuffs during the phases of pre-adaptation, adaptation and finishing on *in situ* degradability of nutrients of cannulated Nellore cattle.

	**Treatments**				
**Item**	**Control**	**Restriction**	**Concentrate**	**SEM**	***P*-value**
**Pre-adaptation (days 6 to 8)**					
Dry matter,%	61.94^b^	64.55^b^	71.25^a^	2.64	<0.01
Neutral detergent fiber,%	37.52	39.31	45.30	5.59	0.56
Acid detergent fiber,%	24.60	26.71	33.49	4.64	0.33
Starch,%	79.93^c^	85.46^b^	93.50^a^	3.80	0.02
Crude protein,%	58.01^ab^	56.88^b^	61.10^a^	3.15	0.05
Ether extract,%	75.95^b^	71.34^b^	80.65^a^	3.19	0.04
Ash,%	75.85	74.04	81.18	2.78	0.16
Total digestible nutrients,%	60.85^b^	63.28^b^	69.89^a^	2.36	<0.01
**Adaptation (days 17 to 19)**					
Dry matter,%	68.36^ab^	65.66^b^	70.39^a^	3.13	0.05
Neutral detergent fiber,%	36.37	32.75	37.76	2.23	0.19
Acid detergent fiber,%	22.59	20.28	24.74	2.00	0.14
Starch,%	86.14^ab^	84.04^b^	89.56^a^	4.15	0.05
Crude protein,%	67.08	67.14	68.12	2.60	0.73
Ether extract,%	80.88	76.57	79.09	2.97	0.50
Ash,%	85.26	84.56	85.60	1.46	0.79
Total digestible nutrients,%	67.85^ab^	65.44^b^	69.94^a^	2.93	0.05
**Finishing (days 29 to 31)**					
Dry matter,%	66.34	67.18	68.25	2.02	0.80
Neutral detergent fiber,%	32.01	32.10	32.27	2.59	1.00
Acid detergent fiber,%	21.98	21.29	20.62	3.04	0.95
Starch,%	82.04^b^	87.50^a^	88.15^a^	2.08	0.05
Crude protein,%	71.67	67.70	64.45	4.03	0.20
Ether extract,%	84.65	81.30	76.66	4.37	0.15
Ash,%	90.62^a^	85.28^b^	86.49^b^	2.09	0.03
Total digestible nutrients,%	66.32	66.74	67.75	2.11	0.88

*^*a*,*b*,*c*^For treatment effect, within a row means without common superscript letter differ (*P* < 0.05).*

**FIGURE 1:**
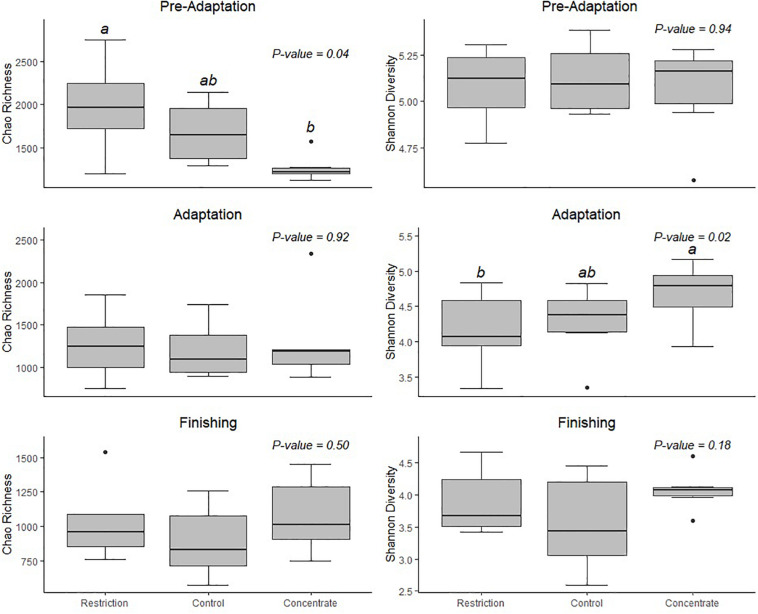
Shannon’s diversity index and Chao’s richness estimator for microbial communities in the rumen of Nellore cattle. Data are expressed as standard boxplots with medians. Outliers are shown as dots. Groups with different letters above the same plot are significantly different (Tukey’s HSD *P* < 0.05).

To better understand the dynamics of the adaptation period, we conducted 16S rRNA microbiota sequencing from ruminal samples collected during the pre-adaptation, adaptation and finishing periods. We generated a total of 152,217 raw sequences, which resulted in an average of 15,956 ± 94 SD sequences per sample that passed filter. The pooled samples contained an average of 3,977 unique OTUs, and a Good’s coverage ≥0.98. We calculated alpha diversity and found that the concentrate-fed group had a higher bacterial diversity than animals in the restriction group in the adaptation phase (Figure 1). The fact that restricted animals had lower DM intake during the adaptation phase, as well as lower digestibility of DM, starch, and total digestible nutrients when compared to cattle consuming concentrate, may explain these observed differences in alpha diversity.

**FIGURE 2:**
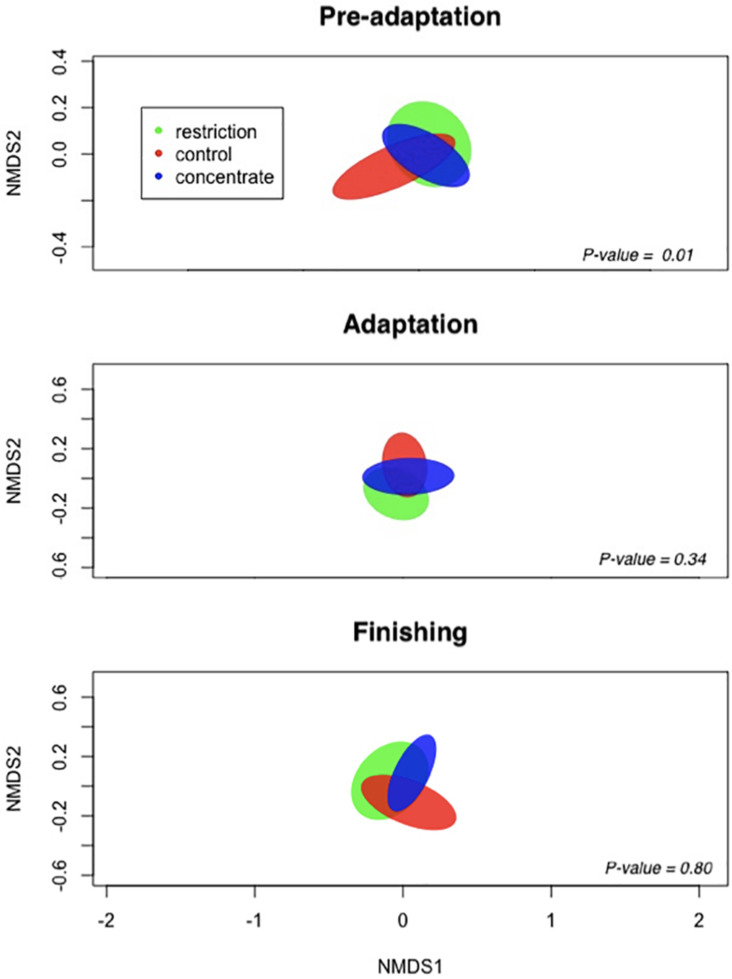
Non-metric multidimensional scaling (NMDS) representation of the Bray–Curtis dissimilarity metric for ruminal content of cannulated Nellore cattle. Ellipses represent 95% confidence intervals of individual samples and are colored by treatment: restriction (green), control (red), and concentrate (blue).

We also note that cattle consuming concentrate had decreased (*P* = 0.04) bacterial richness during the pre-adaptation phase when compared to restricted animals (Figure 1), but this difference was not found for either the adaptation or finishing phases. Likewise, our Bray–Curtis dissimilarity analysis, as visualized using NMDS (Figure 2) revealed a treatment effect (*P* = 0.01) during the pre-adaptation phase, where we found differences in the BCC between rumen content samples from cattle fed the restricted diet and those fed the concentrate or control diet, thereby confirming that nutritional status during the pre-adaptation promotes alterations in the BCC. In contrast, no significant (*P* > 0.05) treatment effect was detected during the adaptation and finishing periods. Taken together, these data suggest that the addition of concentrate to the pre-adaptation diet can alter the ruminal microbiota and facilitate adaptation to a high-concentrate diet. This is supported by our finding that the calculated ruminal bacterial richness of cattle receiving concentrate during the pre-adaptation was as low as the ruminal bacterial richness across treatments during the adaptation and finishing phases (Figure 1).

**FIGURE 3:**
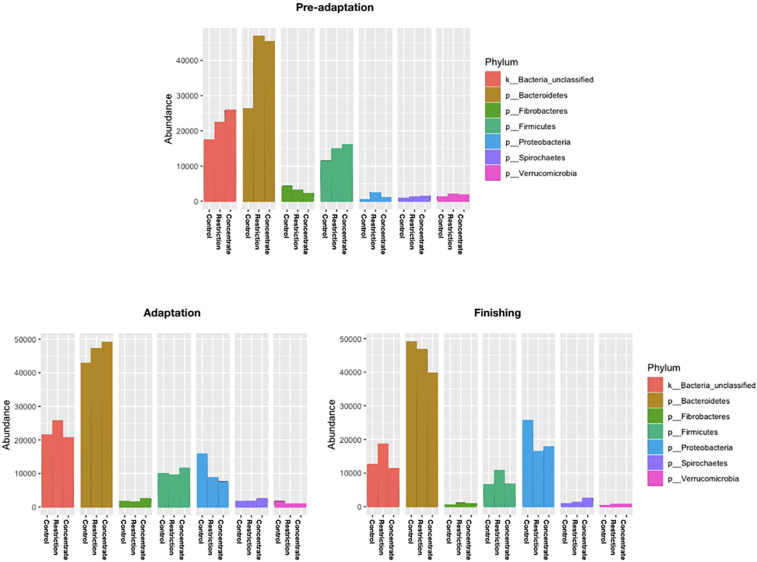
Relative abundance of the top seven phylum of ruminal bacterial communities observed in all cannulated Nellore cattle during pre-adaptation (day 5), adaptation (day 16), and finishing (day 27) in all sampling periods. Samples represent on average ≥ 1% of the total sequence abundance recovered from each animal.

**FIGURE 4:**
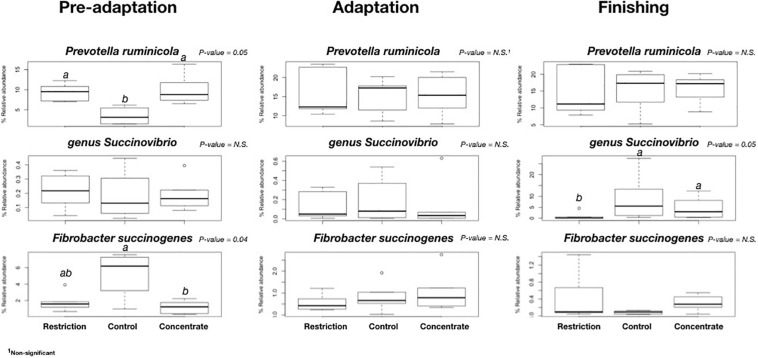
Comparison of relative abundances of bacterial species found to be significantly different in all cannulated Nellore cattle during pre-adaptation (day 5), adaptation (day 16), or finishing (day 27). Only those OTUS with on average ≥ 1% sequence abundance in each animal were considered in this comparison. ^a,b^For treatment effect, within a row means without common superscript letter differ (*P* < 0.05).

We also found no major changes for phylum abundance during the pre-adaptation, adaptation, or finishing phases (Figure 3), and no significant differences (*P* > 0.05) in the abundance of bacterial genera and species during the adaptation phase across treatments (Figure 4). However, it is noteworthy to mention that cattle fed concentrate had lower (*P* = 0.04) relative abundances of *Fibrobacter succinogenes* when compared to animals in the control group during the pre-adaptation phase (Figure 4). This may be related to the larger (*P* = 0.03) area of pH below 6.2 (Supplementary Table S2), which likely resulted in the decrease in total tract digestibility of neutral and ADFs that we also observed (*P* < 0.04; Supplementary Table S6). In the pre-adaptation phase, our correlation analysis identified 7 OTUs correlated with pH, but none of them were found to be significant interactions (data not shown).

For the finishing phase, DM intake was similar between concentrate-fed cattle and those from the control group. However, cattle that were restricted in the pre-adaptation phase still had lesser DM intake than either the concentrate or control group diet. Moreover, cattle exposed to concentrate during the pre-adaptation phase had a smaller (*P* = 0.01) area of pH below 5.2 during the finishing phase when compared to control animals at similar DM intakes (Supplementary Table S5). In the finishing phase, 2 OTUs were found to have strong correlations with pH, but neither were considered significant (data not shown). Pereira et al. (2020) reported that cattle previously exposed to intake of concentrate feedstuffs for 32 days improved feedlot performance and had heavier carcass weight after 112 days on feed, when compared to control animals. Furthermore, the same study noted previous nutritional status did not impact the time required for cattle to adapt to the high-concentrate diet, which lasted, on average, 14 days for all treatments.

Early exposure to concentrate feedstuffs is thought to prepare the ruminal bacterial community for higher levels of non-fibrous carbohydrates (Pereira et al., 2020). Our results support this as those animals fed concentrate during the pre-adaptation phase has an absence of diet particle sorting during the adaptation and finishing periods (Supplementary Table S4) without major changes to their ruminal fermentation patterns (Table 2) and BCC (Figure 3). As a result, these animals exhibited increased ruminal starch degradability (Table 3), which we posit may positively impact the capacity of the rumen epithelium for SCFAs clearance. In particular, Costa et al. (2008) reported that propionate is responsible for promoting active ruminal papillae growth, and based on the results of this study, cattle from the concentrate group had greater (*P* = 0.04) propionate concentration during the adaptation phase, when compared to control animals (Table 2), which may have contributed to greater development of the rumen papillae of those animals (Costa et al., 2008). As a result, concentrate-fed cattle had greater (*P* = 0.05) starch ruminal degradability than control cattle (Table 3), which may be related to the decreased bacterial richness during the pre-adaptation period (Figure 1).

We also found that cattle from the restriction group also had a smaller (*P* = 0.01) area below pH 5.2 when compared to the control group during the finishing phase (Supplementary Table S2). The restriction group also had a lower DM intake (and consequently lower starch intake), which likely led to lower (*P* = 0.04) propionate concentration in the rumen (Table 2) and lower (*P* < 0.01) DM concentration in the rumen (Supplementary Table S2), despite greater ruminal degradability of starch (Table 3). Moreover, cattle from the restriction group had a lower (*P* = 0.05) relative abundance of *Succinivibrio* when compared to cattle from the control and concentrate groups (Figure 4). We also found that 19 OTUs had strong correlations with the propionate concentrations during the finishing phase, but only 2 of these were significant [OTU286, an Unclassified Bacteriodales (*P* = 0.0334) and OTU318 an Unclassified Bacteria (*P* = 0.0110)]. These findings may explain the lower propionate concentration, as members of the *Succinivibrio* and Bacteroidales are known starch degraders in the rumen and found in high abundance when cattle are fed high-grain diets containing large amounts of starch or rapidly fermentable carbohydrates (Zhang et al., 2018). Ren et al. (2019) also reported that the relative abundance of amylolytic *Succinivibrio* increased as the availability of starch in the diet increased, resulting in a positive correlation with propionate concentration in the rumen, which is in agreement with the results observed in this study.

In addition to characterizing the BCC, we also quantified protozoa within the rumens of our cohorts (Table 4). We found that populations of protozoa from the genus *Diplodinium* and *Dasytricha* were greater (*P* ≤ 0.01) for both concentrate-fed and restricted animals when compared to those from the control group during the finishing stage. This was probably a negative effect resulting from the larger (*P* = 0.01) area of pH below 5.2 presented by cattle in the control group during the finishing phase (Supplementary Table S2), since both genera are sensitive to low ruminal pH (Jouany and Ushida, 1999; Franzolin and Dehority, 2010). This is supported by our finding of a strong correlation and significant interaction between numbers of *Diplodinium* and pH levels (*P* = 0.00422). It has been reported that ruminal protozoa play an important role in positively regulating ruminal pH by both predating on ruminal bacteria and engulfing starch granules (Nagaraja and Titgemeyer, 2007). This may help concentrate-fed cattle go through the adaptation and finishing phases without decreasing their DM intake. However, when rumen pH drops below 5.6, the vagus nerve is activated and rumen motility is decreased in an effort to reduce acid production, which can result in lower DM intake (Owens et al., 1998). This may explain why the restricted animals in our study sorted for long and medium diet particles (Supplementary Table S4) in an effort to alleviate rumen acidification.

**TABLE 4:** Effects of either nutritional restriction or intake of concentrate feedstuffs during the phases of pre-adaptation (day 5), adaptation (day 16), and finishing (day 27) on differential counts of ciliated protozoa (10^3^/mL) of cannulated Nellore cattle.

	**Treatments**				
**Item**	**Control**	**Restriction**	**Concentrate**	**SEM**	***P*-value**
**Pre-adaptation (day 5)**					
*Entodinium*	57.50^b^	44.1^b^	99.35^a^	9.92	<0.01
*Diplodinium*	19.40	29.35	21.00	4.60	0.46
*Isotricha*	8.15	6.35	6.05	2.66	0.36
*Dasytricha*	4.55	3.35	2.65	0.74	0.29
Total protozoa	90.25^b^	83.7^b^	129.8^a^	12.62	<0.01
**Adaptation (day 16)**					
*Entodinium*	153.95	165.20	148.80	19.91	0.45
*Diplodinium*	18.65	18.95	13.85	5.71	0.69
*Isotricha*	6.85	3.15	1.60	2.21	0.16
*Dasytricha*	1.65^a^	2.15^a^	1.00^b^	0.47	<0.01
Total protozoa	181.65	189.85	165.1	16.14	0.16
**Finishing (day 27)**					
*Entodinium*	193.60	196.05	207.25	15.53	0.78
*Diplodinium*	13.15^b^	27.5^a^	26.5^a^	9.85	<0.01
*Isotricha*	1.85	0.75	1.55	0.96	0.28
Dasytricha	0.005^b^	0.85^a^	1.15^a^	0.21	0.01
Total protozoa	209.55	226.15	236.95	18.12	0.38

*^*a*,*b*^For treatment effect, within a row means without common superscript letter differ (*P* < 0.05).*

We also found that the inclusion of concentrate feedstuffs in the diets during the pre-adaptation phase increased (*P* < 0.01) populations of protozoa from the genus *Entodinium* (Table 4), which is less sensitive to low ruminal pH (Mackie et al., 1978; Franzolin and Dehority, 2010). This genus is the most dominant protozoan in the rumen when cattle are fed high-concentrate diets and as they can rapidly degrade starch, resulting in faster growth rates (Matthews et al., 2019). During the adaptation and finishing phases, cattle from the restriction and control groups did not have significantly different (*P* > 0.05) *Entodinium* counts when compared to concentrate-fed animals (Table 4).

We then considered if correlations exist between ruminal protozoa and bacteria and found 7 OTUs with strong association with *Diplodinium*, although none of them were significant. A similar analysis considering *Dasytricha* also revealed 9 strong associations with ruminal bacteria, with 2 of these found to be significant: OTU1022 (*P* = 0.0489) and OTU803 (*P* = 0.00643), both of where were identified as Unclassified Bacteria. Given these findings, it is clear that while interactions between ruminal protozoa and bacteria exist, more work should be performed in order to understand their impact on ruminal function.

In summary, despite the treatment effects discussed above, no significant effects of the treatments during the finishing phase were observed on feeding behavior and apparent total tract digestibility of nutrients, which indicates that different nutritional backgrounds may have had their effects diluted over time as cattle are adapted to and finished with the same diets. However, cattle previously exposed to concentrate appear to adapt better to high concentrate finishing diets, since DM intake and ruminal pH were not negatively affected. This is reflected in the ruminal microbiota, as these animals exhibited decreased bacterial richness during the pre-adaptation phase and increased bacterial diversity during the adaptation phase. Thus, major changes in BCC, as well as ruminal fermentation patterns and nutrient digestibility due to the previous nutritional status, indicate that the energy content of the finishing diet could be adjusted according to the nutritional background of cattle, but this deserves further investigation.

## Data Availability Statement

The raw data supporting the conclusions of this article will be made available by the authors, without undue reservation, to any qualified researcher.

## Ethics Statement

The animal study was reviewed and approved by São Paulo State University Ethical Committee for Animal Research (protocol number 05/2015).

## Author Contributions

AP was the master student in charge of all sample collections from rumen fluid to feces, orts, and diets. GB was the master student responsible to adjust the diets daily and feed the animals according to the protocol proposed for this study. LF was responsible for the protozoa counting in rumen samples collected in this study. ED was responsible for the analysis of nutrients in feces samples collected in this study. BD was responsible for the analysis of nutrients in diet samples collected in this study. AN was in charge of all data collection involving feeding behavior and particle sorting. MS was one of the students responsible for emptying the rumens for the ruminal dynamics technique. MS also helped to feed cattle every day and on day-to-day tasks. AS was one of the students responsible for emptying the rumens for the ruminal dynamics technique. AS was also in charge of the cattle during the washout periods. LO was one of the students responsible for emptying the rumens for the ruminal dynamics technique. LO was also in charge of pen’s cleaning during the washout periods. JS was one of the students who assisted in next-generation sequencing of the ruminal microbiota and assisted in the statistical analysis of this study. PR was co-investigator in this study and contributed by coordinating the short-chain fatty acids analysis, as well as by collaborating on experimental design, and data discussion. GC was co-investigator in this study and contributed by collaborating on experimental design and data discussion. GS was co-investigator in this study and contributed by coordinating the next-generation sequencing analysis, and by collaborating on statistical analysis and data discussion. DM was the principal investigator in this study. He trained the grad and undergrad in all techniques used in this study, as well as was trained by GS’s team to perform next-generation sequencing analysis. DM was also in charge of experimental design and statistical analysis of this study. All authors contributed to the article and approved the submitted version.

## Conflict of Interest

GC was employed by Purina Animal Nutrition LLC.

The remaining authors declare that the research was conducted in the absence of any commercial or financial relationships that could be construed as a potential conflict of interest.
