# Supplementary material for: Ruminal Fermentation Pattern, Bacterial Community Composition, and Nutrient Digestibility of Nellore Cattle Submitted to Either Nutritional Restriction or Intake of Concentrate Feedstuffs Prior to Adaptation Period
Source: Front Microbiol. 2020 Jul 31;11:1865. doi: 10.3389/fmicb.2020.01865 (PMC7412545; doi:10.3389/fmicb.2020.01865)
Supplement: Supplementary file 1 [file Table_1.DOCX]

**Table S1.** Feed ingredients and chemical composition of the experimental diets fed to cannulated Nellore cattle during the phases of adaptation and finishing.

| Diets | Adaptation 1 | Adaptation 2 | Finishing |
| --- | --- | --- | --- |
| Level of Concentrate (%) | 72 | 79 | 86 |
| *Ingredients, % of dry matter (****DM****)* |  |  |  |
| Sugarcane bagasse | 14.00 | 10.50 | 7.00 |
| Tifton hay | 14.00 | 10.50 | 7.00 |
| Finely ground corn grain | 51.00 | 60.50 | 73.50 |
| Cottonseed meal (38% CP) | 18.70 | 15.70 | 9.00 |
| Urea | 0.80 | 1.00 | 1.20 |
| Limestone | 0.50 | 0.60 | 0.80 |
| Supplement^1^ | 1.00 | 1.20 | 1.50 |
| *Nutrient Content* |  |  |  |
| Total digestible nutrients, % of DM | 69.00 | 72.00 | 75.00 |
| Net energy for maintenance, Mcal/kg of DM | 1.10 | 1.18 | 1.27 |
| Net energy for gain, Mcal/kg of DM | 18.00 | 14.00 | 10.00 |
| Crude protein, % of DM | 15.00 | 15.00 | 14.10 |
| Neutral detergent fiber (**NDF**), % of DM | 36.80 | 31.30 | 24.70 |
| Ether extract, % of DM | 2.50 | 2.70 | 3.00 |
| Physically effective NDF, % of DM | 27.00 | 23.00 | 19.00 |
| Ca, % of DM | 0.61 | 0.65 | 0.72 |
| P, % of DM | 0.46 | 0.46 | 0.42 |

^1^Ca: 9.80%; P: 4.50%; Mg: 4.40%; K: 6.15%; Na: 11.45%; Cl: 6.60%; S: 4.00%; Co: 48.50 ppm; Cu: 516 ppm; Fe: 30 ppm; Mn: 760 ppm; Se: 9 ppm; Zn: 2516.5 ppm; sodium monensin: 2000 ppm.

Table S2. Effects of either nutritional restriction or intake of concentrate feedstuffs during the phases of pre-adaptation (day 5), adaptation (day 16) and finishing (day 27) on ruminal pH, temperature, and ox-redox potential of cannulated Nellore cattle.

|  | Treatments | | |  |  |
| --- | --- | --- | --- | --- | --- |
| Item | Control | Restriction | Concentrate | SEM | P-value |
| *Pre-adaptation (day 5)* |  |  |  |  |  |
| Mean rumen pH | 6.51^b^ | 6.60^a^ | 6.42^c^ | 0.04 | 0.04 |
| Maximum rumen pH | 6.82 | 6.88 | 6.79 | 0.03 | 0.17 |
| Minimum rumen pH | 6.05^b^ | 6.28^a^ | 5.94^b^ | 0.07 | 0.03 |
| Duration pH < 5.2, min | n.d.a.^1^ | n.d.a. | n.d.a. | n.d.a. | n.d.a. |
| Duration pH < 5.6, min | n.d.a. | n.d.a. | n.d.a. | n.d.a. | n.d.a. |
| Duration pH < 6.2, min | 117.22^b^ | 23.89^b^ | 290.00^a^ | 64.06 | 0.05 |
| Area pH < 5.2, pH×min | n.d.a. | n.d.a. | n.d.a. | n.d.a. | n.d.a. |
| Area pH < 5.6, pH×min | n.d.a. | n.d.a. | n.d.a. | n.d.a. | n.d.a. |
| Area pH < 6.2, pH×min | 13.58^b^ | 1.42^b^ | 70.10^a^ | 18.08 | 0.03 |
| Rumen temperature |  |  |  |  |  |
| Rumen oxi-redox potential |  |  |  |  |  |
| *Adaptation (day 16)* |  |  |  |  |  |
| Mean rumen pH | 6.07 | 5.93 | 6.07 | 0.05 | 0.12 |
| Maximum rumen pH | 6.61^a^ | 6.47^b^ | 6.64^a^ | 0.06 | 0.01 |
| Minimum rumen pH | 5.48 | 5.40 | 5.49 | 0.07 | 0.23 |
| Duration pH < 5.2, min | 1.11 | 73.89 | 2.78 | 36.57 | 0.30 |
| Duration pH < 5.6, min | 229.44 | 301.67 | 145 | 101.3 | 0.25 |
| Duration pH < 6.2, min | 918.89 | 997.78 | 892.78 | 61.73 | 0.41 |
| Area pH < 5.2, pH×min | 0.01 | 11.66 | 0.05 | 6.00 | 0.31 |
| Area pH < 5.6, pH×min | 14.51 | 84.2 | 22.9 | 27.94 | 0.11 |
| Area pH < 6.2, pH×min | 281.35 | 443.43 | 304.15 | 66.94 | 0.11 |
| Rumen temperature |  |  |  |  |  |
| Rumen oxi-redox potential |  |  |  |  |  |
| *Finishing (day 27)* |  |  |  |  |  |
| Mean rumen pH | 5.79 | 5.90 | 5.81 | 0.11 | 0.30 |
| Maximum rumen pH | 6.59^a^ | 6.52^a^ | 6.42^b^ | 0.06 | 0.01 |
| Minimum rumen pH | 5.16 | 5.3 | 5.23 | 0.11 | 0.28 |
| Duration pH < 5.2, min | 140 | 51.11 | 52.78 | 53.49 | 0.07 |
| Duration pH < 5.6, min | 463.89 | 313.89 | 496.67 | 144.92 | 0.20 |
| Duration pH < 6.2, min | 1167.2 | 1110.56 | 1133.33 | 117.34 | 0.81 |
| Area pH < 5.2, pH×min | 19.76^a^ | 3.71^b^ | 7.63^b^ | 7.50 | 0.01 |
| Area pH < 5.6, pH×min | 135.46 | 70.77 | 108.16 | 46.6 | 0.14 |
| Area pH < 6.2, pH×min | 631.07 | 486.95 | 599.59 | 134.34 | 0.24 |
| Rumen temperature |  |  |  |  |  |
| Rumen oxi-redox potential |  |  |  |  |  |

^1^No data available.

Table S3. Effects of either nutritional restriction or intake of concentrate feedstuffs during the phases of pre-adaptation, adaptation and finishing on disappearance rate and ruminal dynamics of cannulated Nellore cattle.

|  | Treatments | | |  |  |
| --- | --- | --- | --- | --- | --- |
| Item | Control | Restriction | Concentrate | SEM | P-value |
| *Pre-adaptation (days 9 and 10)* |  |  |  |  |  |
| Solid mass, kg | 5.38^a^ | 4.60^b^ | 4.47^b^ | 0.32 | <0.01 |
| Liquid mass, kg | 35.24^a^ | 33.32^b^ | 29.41^c^ | 2.36 | <0.01 |
| Total mass, kg | 40.62^a^ | 37.92^b^ | 33.89^c^ | 2.68 | <0.01 |
| Solid mass, % PV¹ | 2.05^a^ | 1.70^b^ | 1.71^b^ | 0.09 | <0.01 |
| Liquid mass, % PV | 13.34^a^ | 12.39^b^ | 11.20^c^ | 0.40 | <0.01 |
| Total mass, % PV | 15.38^a^ | 14.10^b^ | 12.91^c^ | 0.47 | <0.01 |
| Dry matter Kt^2^ , %/h | 3.82^b^ | 3.80^b^ | 5.33^a^ | 0.24 | <0.01 |
| Solid Kt^2^, kg/h | 0.21^b^ | 0.17^c^ | 0.24^a^ | 0.01 | <0.01 |
| Dry matter of rumen content, % | 13.27^a^ | 12.06^b^ | 13.28^a^ | 0.34 | 0.03 |
| *Adaptation (days 20 and 21)* |  |  |  |  |  |
| Solid mass, kg | 4.12 | 4.22 | 3.98 | 0.32 | 0.25 |
| Liquid mass, kg | 24.06 | 25.31 | 24.72 | 2.23 | 0.49 |
| Total mass, kg | 28.18 | 29.53 | 28.70 | 2.55 | 0.49 |
| Solid mass, % PV¹ | 1.49 | 1.52 | 1.46 | 0.06 | 0.38 |
| Liquid mass, % PV | 8.68 | 9.08 | 9.05 | 0.44 | 0.42 |
| Total mass, % PV | 10.16 | 10.60 | 10.51 | 0.50 | 0.47 |
| Dry matter Kt^2^ , %/h | 7.77^b^ | 7.61^b^ | 8.49^a^ | 0.32 | 0.03 |
| Solid Kt^2^, kg/h | 0.32^b^ | 0.32^b^ | 0.34^a^ | 0.03 | 0.05 |
| Dry matter of rumen content, % | 14.58 | 14.34 | 13.99 | 0.32 | 0.25 |
| *Finishing (days 32 and 33)* |  |  |  |  |  |
| Solid mass, kg | 3.63 | 3.97 | 3.81 | 0.43 | 0.38 |
| Liquid mass, kg | 19.86^b^ | 23.67^a^ | 21.80^ab^ | 2.28 | 0.05 |
| Total mass, kg | 23.49^b^ | 27.63^a^ | 25.61^ab^ | 2.73 | 0.05 |
| Solid mass, % PV¹ | 1.21 | 1.28 | 1.24 | 0.07 | 0.74 |
| Liquid mass, % PV | 6.62^c^ | 7.65^a^ | 7.15^b^ | 0.23 | 0.02 |
| Total mass, % PV | 7.82^c^ | 8.93^a^ | 8.38^b^ | 0.28 | 0.03 |
| Dry matter Kt^2^ , %/h | 9.87 | 8.65 | 8.70 | 1.69 | 0.37 |
| Solid Kt^2^, kg/h | 0.34 | 0.33 | 0.33 | 0.07 | 0.84 |
| Dry matter of rumen content, % | 15.33^a^ | 14.28^b^ | 14.80^ab^ | 0.88 | <0.01 |

^1^Body weight; ^2^ Disappearance rate.

Table S4. Effects of either nutritional restriction or intake of concentrate feedstuffs during the phases of pre-adaptation (day 4), adaptation (day 15) and finishing (day 26) on particle sorting of cannulated Nellore cattle.

|  | Treatments | | |  |  |
| --- | --- | --- | --- | --- | --- |
| Item | Control | Restriction | Concentrate | SEM | P-value |
| *Pre-adaptation (day 4)* |  |  |  |  |  |
| Long | 1.08^a^ | 1.00^b^ | 1.08^a^ | 0.02 | <0.01 |
| Medium | 1.03^a^ | 1.00^ab^ | 0.96^b^ | 0.04 | 0.05 |
| Short | 0.99 | 1.00 | 0.98 | 0.01 | 0.12 |
| Fine | 0.86^b^ | 0.98^a^ | 0.79^b^ | 0.04 | 0.01 |
| *Adaptation (day 15)* |  |  |  |  |  |
| Long | 0.89^b^ | 1.21^a^ | 0.98^b^ | 0.09 | 0.05 |
| Medium | 0.99^b^ | 1.22^a^ | 1.03^b^ | 0.04 | <0.01 |
| Short | 1.04^a^ | 1.05^a^ | 1.01^b^ | 0.01 | 0.02 |
| Fine | 1.03^a^ | 0.82^b^ | 1.06^a^ | 0.34 | 0.05 |
| *Finishing (day 26)* |  |  |  |  |  |
| Long | 1.16 | 1.08 | 1.09 | 0.03 | 0.13 |
| Medium | 1.04 | 1.07 | 1.07 | 0.03 | 0.36 |
| Short | 1.03 | 1.03 | 1.03 | 0.02 | 0.84 |
| Fine | 0.98 | 0.93 | 0.94 | 0.03 | 0.37 |

Table S5. Effects of either nutritional restriction or intake of concentrate feedstuffs during the phases of pre-adaptation (day 4), adaptation (day 15) and finishing (day 26) on feeding behavior of cannulated Nellore cattle.

|  | Treatments | | | |  |  |
| --- | --- | --- | --- | --- | --- | --- |
| Item | Control | Restriction | Concentrate | SEM | | P-value |
| *Pre-adaptation (day 4)* |  |  |  |  | |  |
| Time spent resting, min | 591.67^b^ | 715.00^a^ | 624.17^b^ | 41.57 | | 0.04 |
| Time spent ruminating, min | 477.50 | 420.00 | 461.67 | 25.93 | | 0.14 |
| Time spent eating, min | 353.33^a^ | 287.50^b^ | 342.50^a^ | 23.93 | | 0.03 |
| Meal length, min | 25.57^a^ | 24.76^a^ | 18.75^b^ | 1.97 | | 0.01 |
| Meals per day, n | 14.17^b^ | 12.00^b^ | 18.33^a^ | 1.11 | | <0.01 |
| Daily DM^1^ intake, kg | 5.25^a^ | 3.56^b^ | 4.85^a^ | 0.45 | | 0.02 |
| DM intake per meal, kg | 0.38^a^ | 0.31^b^ | 0.27^b^ | 0.04 | | 0.01 |
| ER^2^ of DM, min/kg of DM | 69.55 | 81.19 | 73.56 | 10.44 | | 0.16 |
| RR^3^ of DM, min/kg of DM | 92.36^b^ | 118.11^a^ | 98.74^b^ | 8.90 | | 0.02 |
| NDF^4^ intake, kg | 4.08^a^ | 2.73^c^ | 3.63^b^ | 0.29 | | <0.01 |
| ER of NDF, min/kg of DM | 89.08 | 106.04 | 97.00 | 12.68 | | 0.11 |
| RR of NDF, min/kg of DM | 117.99^b^ | 154.28^a^ | 130.84^b^ | 10.02 | | 0.01 |
| *Adaptation (day 15)* |  |  |  |  | |  |
| Time spent resting, min | 729.17^ab^ | 781.67^a^ | 695.00^b^ | 43.51 | | 0.04 |
| Time spent ruminating, min | 437.50 | 405.00 | 451.67 | 34.94 | | 0.18 |
| Time spent eating, min | 258.33 | 237.50 | 270.00 | 25.21 | | 0.23 |
| Meal length, min | 17.14 | 15.61 | 19.23 | 2.02 | | 0.18 |
| Meals per day, n | 16.17 | 16.17 | 15.50 | 2.36 | | 0.91 |
| Daily DM^1^ intake, kg | 5.09^a^ | 4.23^b^ | 5.46^a^ | 0.49 | | <0.01 |
| DM intake per meal, kg | 0.35^ab^ | 0.30^b^ | 0.42^a^ | 0.08 | | 0.05 |
| ER^2^ of DM, min/kg of DM | 52.85 | 57.56 | 53.39 | 8.82 | | 0.47 |
| RR^3^ of DM, min/kg of DM | 88.84 | 98.49 | 89.68 | 14.24 | | 0.39 |
| NDF^4^ intake, kg | 2.69 | 2.23 | 2.56 | 0.42 | | 0.36 |
| ER of NDF, min/kg of DM | 119.06 | 111.98 | 127.32 | 26.84 | | 0.85 |
| RR of NDF, min/kg of DM | 202.11 | 190.18 | 210.97 | 51.77 | | 0.89 |
| *Finishing (day 26)* |  |  |  |  | |  |
| Time spent resting, min | 855.83 | 884.17 | 855.17 | 22.25 | | 0.14 |
| Time spent ruminating, min | 349.17 | 337.50 | 345.83 | 25.76 | | 0.81 |
| Time spent eating, min | 214.17 | 199.17 | 214.17 | 12.70 | | 0.32 |
| Meal length, min | 12.86 | 14.09 | 12.83 | 1.22 | | 0.46 |
| Meals per day, n | 17.67 | 15.17 | 17.00 | 2.09 | | 0.15 |
| Daily DM^1^ intake, kg | 5.83 | 5.99 | 5.87 | 0.58 | | 0.79 |
| DM intake per meal, kg | 0.37 | 0.43 | 0.36 | 0.08 | | 0.39 |
| ER^2^ of DM, min/kg of DM | 39.30 | 33.83 | 37.47 | 5.58 | | 0.23 |
| RR^3^ of DM, min/kg of DM | 61.97 | 57.74 | 60.63 | 10.00 | | 0.43 |
| NDF^4^ intake, kg | 2.13 | 2.16 | 2.07 | 0.17 | | 0.86 |
| ER of NDF, min/kg of DM | 106.57 | 94.57 | 105.61 | 12.99 | | 0.50 |
| RR of NDF, min/kg of DM | 173.89 | 159.28 | 168.54 | 21.54 | | 0.65 |

^1^Dry matter; ^2^Eating rate; ^3^Rumination rate; ^4^Neutral detergent fiber;

Table S6. Effects of either nutritional restriction or intake of concentrate feedstuffs during pre-adaptation and finishing on apparent total tract digestibility of nutrients of cannulated Nellore cattle.

|  | Treatments | | |  |  |
| --- | --- | --- | --- | --- | --- |
| Item | Control | Restriction | Concentrate | SEM | P-value |
| *Pre-adaptation (days 10 to 14)* |  |  |  |  |  |
| Organic matter, % | 59.78^a^ | 52.38^b^ | 60.26^a^ | 2.22 | <0.01 |
| Dry matter, % | 57.26^a^ | 49.82^b^ | 58.48^a^ | 1.99 | <0.01 |
| Neutral detergent fiber, % | 62.43^a^ | 56.68^b^ | 55.71^b^ | 2.80 | 0.04 |
| Acid detergent fiber, % | 61.85^a^ | 58.02^a^ | 48.64^b^ | 3.30 | 0.03 |
| Starch, % | 75.67^b^ | 62.07^c^ | 86.90^a^ | 6.83 | <0.01 |
| Crude protein, % | 57.33^a^ | 47.56^b^ | 56.84^a^ | 2.37 | <0.01 |
| Ether extract, % | 58.56^a^ | 32.58^b^ | 50.87^a^ | 4.63 | <0.01 |
| Ash, % | 18.17^b^ | 13.59^c^ | 31.35^a^ | 3.03 | <0.01 |
| Total digestible nutrients, % | 58.01^a^ | 49.75^b^ | 57.75^a^ | 2.08 | <0.01 |
| *Finishing (days 29 to 33)* |  |  |  |  |  |
| Organic matter, % | 70.05 | 70.01 | 69.78 | 2.01 | 0.99 |
| Dry matter, % | 68.47 | 68.41 | 68.34 | 2.01 | 1.00 |
| Neutral detergent fiber, % | 57.82 | 56.15 | 55.52 | 3.66 | 0.90 |
| Acid detergent fiber, % | 51.81 | 44.21 | 42.73 | 6.83 | 0.96 |
| Starch, % | 87.64 | 88.57 | 87.31 | 1.52 | 0.71 |
| Crude protein, % | 72.59 | 72.25 | 72.69 | 1.77 | 0.98 |
| Ether extract, % | 78.02 | 80.80 | 80.89 | 5.37 | 0.91 |
| Ash, % | 41.15 | 39.63 | 42.20 | 3.76 | 0.89 |
| Total digestible nutrients, % | 69.53 | 69.39 | 68.94 | 2.08 | 0.97 |
